# Visualization of expanding warm dense gold and diamond heated rapidly by laser-generated ion beams

**DOI:** 10.1038/srep14318

**Published:** 2015-09-22

**Authors:** W. Bang, B. J. Albright, P. A. Bradley, D. C. Gautier, S. Palaniyappan, E. L. Vold, M. A. Santiago Cordoba, C. E. Hamilton, J. C. Fernández

**Affiliations:** 1 Los Alamos National Laboratory, Los Alamos, New Mexico 87545, USA

## Abstract

With the development of several novel heating sources, scientists can now heat a small sample isochorically above 10,000 K. Although matter at such an extreme state, known as warm dense matter, is commonly found in astrophysics (e.g., in planetary cores) as well as in high energy density physics experiments, its properties are not well understood and are difficult to predict theoretically. This is because the approximations made to describe condensed matter or high-temperature plasmas are invalid in this intermediate regime. A sufficiently large warm dense matter sample that is uniformly heated would be ideal for these studies, but has been unavailable to date. Here we have used a beam of quasi-monoenergetic aluminum ions to heat gold and diamond foils uniformly and isochorically. For the first time, we visualized directly the expanding warm dense gold and diamond with an optical streak camera. Furthermore, we present a new technique to determine the initial temperature of these heated samples from the measured expansion speeds of gold and diamond into vacuum. We anticipate the uniformly heated solid density target will allow for direct quantitative measurements of equation-of-state, conductivity, opacity, and stopping power of warm dense matter, benefiting plasma physics, astrophysics, and nuclear physics.

Acceleration of ions with an intense short laser pulse has been an active area of research in laser-plasma physics over the past two decades^1^,^2^,^3^,^4^,^5^,^6^,^7^,^8^,^9^,^10^,^11^,^12^,^13^,^14^. With the development of these new ion sources, several types of laser-generated ion beams are available for use in applications such as proton radiography^4^, neutron beam generation^15^, fast ignition^16^,^17^, and the rapid heating of a target^18^,^19^,^20^,^21^,^22^,^23^,^24^,^25^. When such ion beams are used to heat a cold target, the energetic ions can transfer a significant amount of their kinetic energy to the target. This heating occurs so quickly (<50 ps) that the target does not have time to expand (isochoric heating).

In isochoric heating experiments with laser-driven ions^18^,^19^,^20^,^21^,^22^,^23^,^24^,^25^, the temperature of the target increases beyond 10,000 K while still maintaining near-solid density, and the originally cold target becomes warm dense matter^26^. With laser-generated ions, one has the potential for high volumetric heating uniformity compared with direct laser heating, where most of the laser light is first absorbed at the front surface of the target (within 100 nm), and the rest of the target is subsequently heated by the resulting hot and return electron currents. Still, uniform heating across the whole target depth is not reached when using ions with a typical exponential energy spectrum because low energy ions tend to heat the front surface of the target more strongly^6^,^20^,^21^,^22^,^23^.

We find that this issue can be resolved by the use of ion beams with a quasi-monoenergetic energy spectrum^5^,^7^,^27^,^28^,^29^,^30^,^31^. Recent work by Palaniyappan *et al.*^31^ reports on the generation of Al^11+^ ion beams with 7–30% energy spread and 5% laser-energy-to-ion-energy conversion efficiency, obtained using ultrathin (110 nm) aluminum foils as laser targets. With such quasi-monoenergetic ion beams, it is possible to select a proper target thickness to avoid most ions from ranging out within the sample so that the incident ions deposit their kinetic energy uniformly throughout the target.

Once a cold solid target is heated isochorically by energetic ions, it expands adiabatically into a vacuum^32^ and this expansion can, in principle, be recorded. Visualization of this expansion, however, has been very challenging primarily owing to geometric constraints. Here we report for the first time on the optical visualization of expanding warm dense gold and diamond heated uniformly and isochorically. Using the ion acceleration technique described in ref. 31, we generated quasi-monoenergetic Al^11+^ ions and heated both gold and diamond targets isochorically to a state of warm dense matter, and used an optical streak camera to record the expansion of gold and diamond into a vacuum. According to our radiation-hydrodynamic simulations with the RAGE code^33^ utilizing SESAME equation-of-state (EOS) tables^34^, the inferred plasma temperatures obtained from the expansion speeds of gold and diamond correspond to 5.5 (±0.5) eV and 1.7 (±0.1) eV, respectively.

## Results

**Figure 1:**
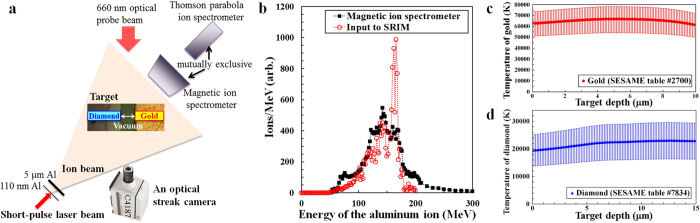
Expected heating uniformity from the measured ion energy spectrum. (**a**) Schematic layout of the experimental setup (not to scale, drawn by W. Bang). (**b**) Measured energy spectrum of the incident aluminum ions along with the input energy spectrum used in SRIM calculations. (**c**) Calculated temperature of gold immediately after being heated by the quasi-monoenergetic aluminum ion beam at a source-to-target distance of 2.37 mm. (**d)** Calculated temperature of diamond immediately after the isochoric heating.

We performed the experiments on the Trident laser facility at Los Alamos National Laboratory (LANL), which delivered 80 J, 650 fs, 1054 nm wavelength pulses to irradiate 110 nm thick aluminum foils. Figure 1a shows the schematic layout of the experimental setup. Utilizing an f/3 off-axis-parabola, the peak intensity of the laser pulse on the thin aluminum foil was 2 × 10^20^ W/cm^2^. The laser-driven aluminum ion beam diverged with a 20° cone half-angle^31^, and impinged upon gold and diamond foils located 2.37 mm from the ion source inside the vacuum target chamber. This source-to-target separation between ion source and target was sufficient to allow for imaging of the expanding warm dense matter. A 5 μm thick aluminum filter, inserted 0.37 mm behind the source and 2.0 mm before the target, blocked any laser light that propagated through the 110 nm aluminum foil after it became relativistically transparent^35^, ensuring the target was indeed heated isochorically with the laser-generated aluminum ion beam.

The ion beam is incident on the target at 45° so that a 660 nm laser beam could be sent from behind the target to probe the locations of the critical surfaces of gold and diamond. The light not blocked by gold or diamond plasma goes into the streak camera (Hamamatsu C4187), showing the edge location of gold and diamond as a function of time. The aluminum ions that go through the vacuum gap between the foils are recorded on the magnetic ion spectrometer^36^, which monitors shot-to-shot fluctuations in the incident ion energy spectra and fluence.

Figure 1b shows the on-shot measurement (from this experiment) of the energy spectrum of the incident aluminum ions along with the input data for a Monte Carlo simulation code, SRIM^37^,^38^. Hollow red circles indicate the energy spectrum used in SRIM to calculate ion stopping powers within the target, based on Thomson parabola ion spectrometer measurements^31^. Solid black squares represent the measured energy spectrum in this experiment, which exhibit the quasi-monoenergetic feature. Since the magnetic ion spectrometer cannot distinguish different charge states, we have used the Thomson parabola measurement for SRIM. The close agreement between the two measurements above 100 MeV in Fig. 1b justifies the use of the Thomson parabola measurement for SRIM simulations.

The SRIM simulations indicate that the quasi-monoenergetic aluminum ion beam heats the target foils very uniformly, a result of a balance between heating from ions in the low-energy part of the spectra absorbed in the target (decreasing stopping power with target depth) and heating from ions from the high-energy part (increasing stopping power). Figure 1c,d show the expected heating uniformity within gold (red circles) and diamond (blue triangles) foils immediately after the isochoric heating. Figure 1c,d show the calculated temperatures of gold and diamond, respectively, as functions of the target depth. The vertical error bars represent the uncertainties in the expected temperatures owing to the observed shot-to-shot fluctuations of ±30% in the incident aluminum ion fluence.

**Figure 2:**
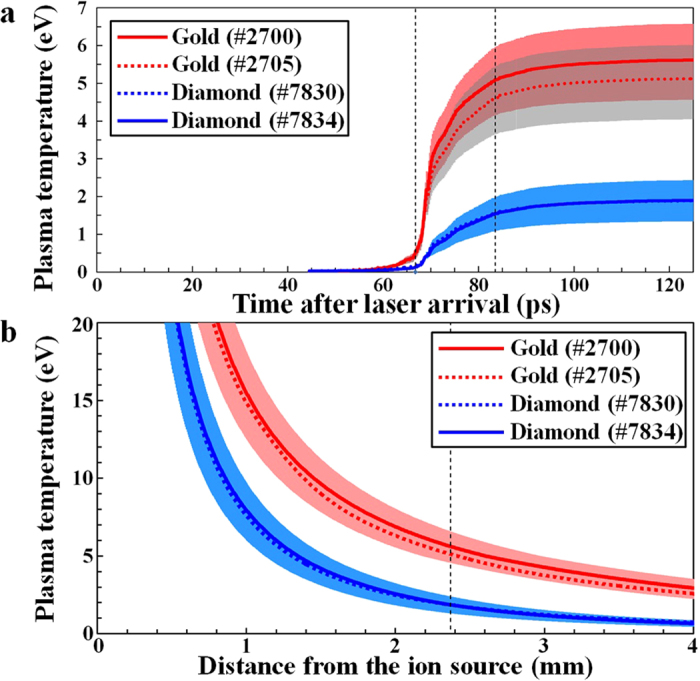
Expected plasma temperatures of rapidly heated gold and diamond. (**a**) The mean plasma temperatures of gold and diamond as functions of time after laser arrival. For gold, SESAME #2700 (solid red line) and #2705 (dashed red line) tables predict slightly different plasma temperatures, while both SESAME #7830 (dashed blue line) and #7834 (solid blue line) tables predict nearly the same plasma temperature for diamond. (**b**) Expected plasma temperatures of gold and diamond as functions of the source-to-target distance, evaluated using heating per atom calculations and their corresponding SESAME tables. A vertical dashed line indicates the distance (2.37 mm) used in this experiment.

Ions with different kinetic energy arrive at the target foils at different times. It takes 125 ps for 50 MeV aluminum ions to travel the source-to-target distance of 2.37 mm, while it only takes 63 ps for 200 MeV ions to travel the same distance. Knowing the stopping powers for aluminum ions with different kinetic energy from SRIM, one can calculate the deposited energy into the target atoms as a function of time using the measured energy spectra in Fig. 1b. We have calculated the deposited energy per gold and diamond atoms as functions of time, and have used the corresponding SESAME EOS tables to calculate the expected temperatures at each time step. Figure 2a shows the calculated mean plasma temperatures (1 eV = 11,600 K) of gold and diamond as functions of time, where *t* = 0 ps is the time of laser arrival at the 110 nm Al foil (=ion source). Two vertical dashed black lines are drawn to illustrate the rise time (=16.7 ps) of heating for gold (#2705) to change from 10% to 90% of the final temperature (=5.1 (±1.0) eV). A similar calculation for diamond (#7834) shows a rise time of 22.1 ps. In both cases, most of the heating occurs during the 65–90 ps interval.

For gold, we have used SESAME tables #2700 and #2705^39^, and the resulting temperatures at the end of heating differ by 0.5 eV. The ±30% shot-to-shot fluctuation in the ion fluence results in error bars of up to ±1.0 eV in the expected temperatures of gold in Fig. 2a, whereas the error bars for diamond are up to ±0.5 eV in the same figure. For diamond, both curves using SESAME tables #7830^40^ and #7834 are in good agreement.

Using the calculated heating per atom [see Supplementary Fig. 2 for details] and SESAME EOS tables for gold (#2700 and #2705) and diamond (#7830 and #7834), the expected plasma temperatures immediately after heating are evaluated as functions of the source-to-target distance in Fig. 2b. The diamond EOS tables result in very similar temperatures, while the gold EOS tables show slight differences. Figure 2b shows that gold reaches higher temperature than diamond in our experimental setup.

Based on calculations of electron-electron and electron-ion collision frequencies within our target, we expect that local thermal equilibrium is reached within several picoseconds after heating^41^. Therefore, the plasma temperatures in Fig. 2a,b represent both the electron and ion temperatures. On the other hand, global thermal equilibrium is not expected to be reached within 10 μm thick gold or 15 μm thick diamond even after several hundreds of nanoseconds from heating, which explains why the initial heating uniformity evidenced in Fig. 1c,d is important for this type of experiment.

**Figure 3:**
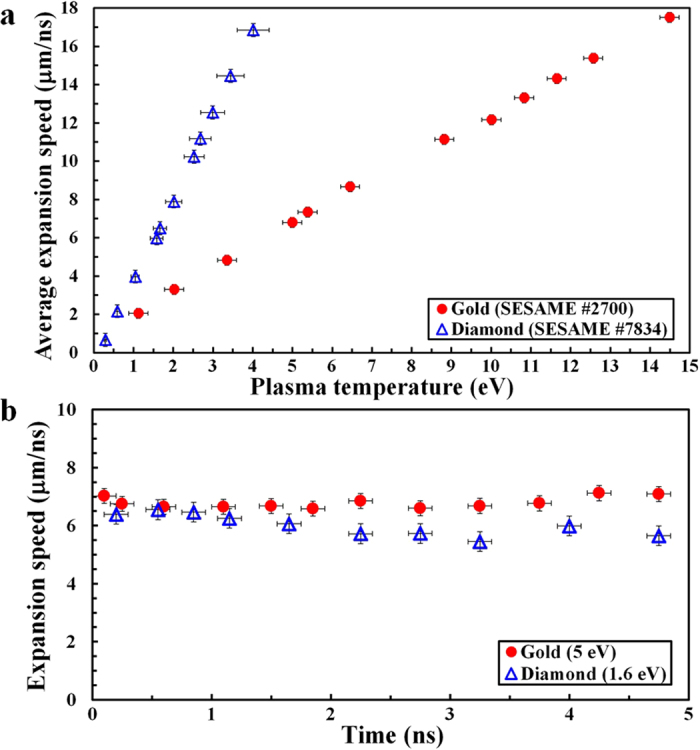
Expansion speeds from 2D RAGE simulations. (**a**) Average expansion speeds of gold (solid red circles) and diamond (hollow blue triangles) into a vacuum as functions of the initial plasma temperature from 2D RAGE simulations. (**b**) Expansion speeds of gold (solid red circles) at 5.0 eV and diamond (hollow blue triangles) at 1.6 eV as functions of time.

Figure 3a,b show the expansion speeds of isochorically heated gold and diamond from RAGE simulations. RAGE is a multi-dimensional, adaptive-mesh-refinement, Eulerian, radiation-hydrodynamic code^33^. Since RAGE lacks ion beam energy deposition, we used a two-dimensional (2D) energy source to mimic the nearly instantaneous ion beam deposition. To compare with experimentally measured optical streak camera images, we created electron density maps from RAGE output and determined the locations of critical density surfaces of gold and diamond for 660 nm light (ray-trace estimates suggest negligible refraction). The expansion speeds of gold and diamond during each time step were calculated, and the time-averaged speed is shown for different initial plasma temperatures of gold and diamond in Fig. 3a. For 5.0 eV gold and 1.6 eV diamond, the expansion speeds are shown as functions of time in Fig. 3b from 0 to 5 ns. Gold expands at 6.8 (±0.3) μm/ns, while diamond expands at 6.0 (±0.3) μm/ns during the first 5 ns after heating. RAGE simulations show the expansion speed stayed nearly constant during this time and was a function of the plasma temperature immediately after heating.

**Figure 4:**
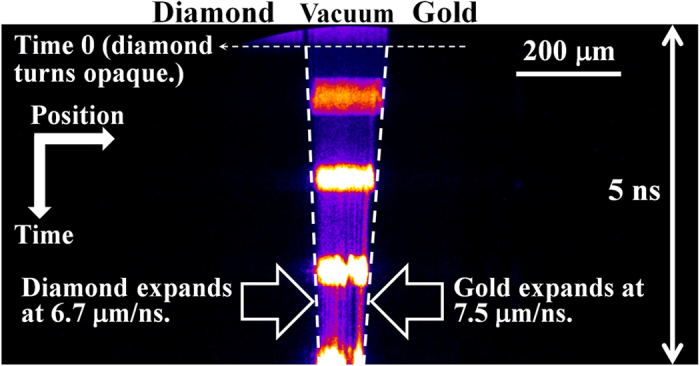
Streak camera image of the expanding gold and diamond into a vacuum. At time 0, the quasi-monoenergetic aluminum ions heat both gold and diamond isochorically, triggering them to expand into a vacuum. The gold expands at 7.5 (±0.6) μm/ns, while the diamond expands at 6.7 (±0.5) μm/ns. The spatial resolution of 2.5–3.0 μm in the image is responsible for the errors in the measured expansion speeds. Note that the diamond turns opaque when it becomes a plasma.

In Fig. 4, the measured streak camera image shows both gold [on the right—see Fig. 1a] and diamond (on the left) expanding into a vacuum (bright region in the middle) after heating. The streaked image records the transmitted 660 nm probe light as a function of time, where time increases in the vertically downward direction. The image spans 5 ns in time, and the time marks (separated by 1.0 ns) imprinted in Fig. 4 confirm the time calibration independently. When the edge of gold or diamond does not move in time, a vertical line is seen in the image, which was confirmed in pre-shot images. A diamond foil transmits 69% of the probe light prior to heating, while a gold foil completely blocks the light. At time 0 (the arrival time of the ions), the ion beam heats both gold and diamond, triggering their expansion into the vacuum. As the diamond becomes a plasma immediately upon heating, it turns opaque to the probe light [see Supplementary Fig. 3, which shows this transition better]. The gold remains opaque both before and after heating.

Both gold and diamond begin to expand after heating, depicted as dashed white lines in Fig. 4. The average expansion speed of gold is 7.5 (±0.6) μm/ns from the image, and RAGE simulations in Fig. 3a indicate that the corresponding temperature of gold plasma immediately after heating is 5.5 (±0.5) eV. This is consistent with the expected temperature of 5.6 (±1.0) eV (SESAME #2700, see Fig. 1c) or 5.1 (±1.0) eV (SESAME #2705) from stopping power calculations with SRIM in Fig. 2b at 2.37 mm. Likewise, the average expansion speed of diamond is 6.7 (±0.5) μm/ns in Fig. 4, and the corresponding plasma temperature is 1.7 (±0.1) eV from Fig. 3a. This temperature also agrees with the expected temperature of 1.9 (±0.5) eV from Fig. 2b at 2.37 mm.

## Discussion

In Fig. 2b, the targets were assumed to be at solid densities immediately after heating because the volume changes during heating were expected to be small. This is a valid assumption considering the following estimate of the volume increase during heating. Figure 2a shows that the heating takes about 20 ps at a source-to-target distance of 2.37 mm. Using the average expansion speed measured from Fig. 4, the volume change during 20 ps is expected to be tiny. For example, a 10 μm thick gold foil becomes 10.3 μm thick after heating because it will expand both ways at an expansion speed as high as 7.5 μm/ns. Since the foil is wide (~1 mm) and high (>1 mm) in the other two dimensions, the volume increase during heating is at most 3%. Similarly, a 15 μm thick diamond can become as thick as 15.3 μm, resulting in a volume increase of 2%. Indeed, the ion beam heats the gold and diamond foils nearly isochorically.

Although the source-to-target distance used in this experiment is about ten times the distances used in earlier isochoric heating experiments^18^,^19^,^20^,^21^,^22^,^23^,^24^, the aluminum ion beam was sufficiently intense to heat gold and diamond isochorically to several eV. In this experiment, we needed sufficient separation for another optical probe beam to have a clear view of both the gold and diamond targets, but we may be able to reduce the source-to-target distance for applications (e.g., off-Hugoniot EOS measurements) where higher target temperatures are desired. At a source-to-target distance of 200 μm, for example, we calculate that we can heat gold up to 100 eV and diamond up to 90 eV with the same ion beam. When lower target temperatures are desired, one can simply increase the source-to-target distance. At a source-to-target distance of 1.0 cm, we can heat gold and diamond foils uniformly to 7,000 K and 2,000 K, respectively, with a 100 ps rise time.

In summary, we have presented results from experiments in which laser-driven aluminum ion beams heated both gold and diamond targets rapidly (~20 ps) to 5.5 eV and 1.7 eV, respectively, in a single shot. Our calculations in Fig. 1c,d based on the measured ion energy spectrum predict good heating uniformity within both targets. An optical streak camera visualized directly the expanding warm dense gold and diamond. For the first time, we observed a direct one-to-one relationship between the time-averaged expansion speed and the initial temperature of warm dense matter. The temperatures of gold and diamond immediately after heating were determined by matching the measured expansion speeds from the streaked image with 2D RAGE simulations, which is a new technique. For both gold and diamond independently, these plasma temperatures agreed well with the expected temperatures computed using stopping power calculations and SESAME EOS tables.

We expect these uniformly heated solid density targets would be useful for EOS, conductivity, and opacity measurements. Astrophysicists may also find such targets to be useful for validating their understanding of the conditions of giant planet interiors^42^. The uniformly heated target can be directly used for stopping power measurements^14^ of warm dense matter, benefiting nuclear physics, too.

## Additional Information

**How to cite this article**: Bang, W. *et al.* Visualization of expanding warm dense gold and diamond heated rapidly by laser-generated ion beams. *Sci. Rep.* **5**, 14318; doi: 10.1038/srep14318 (2015).

## Supplementary Material

Supplementary Information

## References

[b1] Maksimchuk A., Gu S., Flippo K., Umstadter D. & Bychenkov V. Y. Forward ion acceleration in thin films driven by a high-intensity laser. Phys. Rev. Lett. 84, 4108–4111 (2000). 10990622 10.1103/PhysRevLett.84.4108

[b2] Snavely R. *et al.* Intense high-energy proton beams from petawatt-laser irradiation of solids. Phys. Rev. Lett. 85, 2945–2948 (2000). 11005974 10.1103/PhysRevLett.85.2945

[b3] Wilks S. C. *et al.* Energetic proton generation in ultra-intense laser–solid interactions. Phys. Plasmas 8, 542–549 (2001).

[b4] Roth M. *et al.* Energetic ions generated by laser pulses: A detailed study on target properties. Phys. Rev. ST Accel. Beams 5, 061301 (2002).

[b5] Hegelich B. M. *et al.* Laser acceleration of quasi-monoenergetic Mev ion beams. Nature 439, 441–444 (2006). 16437109 10.1038/nature04400

[b6] Schollmeier M. *et al.* Controlled transport and focusing of laser-accelerated protons with miniature magnetic devices. Phys. Rev. Lett. 101, 055004 (2008). 18764401 10.1103/PhysRevLett.101.055004

[b7] Jung D. *et al.* Monoenergetic ion beam generation by driving ion solitary waves with circularly polarized laser light. Phys. Rev. Lett. 107, 115002 (2011). 22026679 10.1103/PhysRevLett.107.115002

[b8] Bartal T. *et al.* Focusing of short-pulse high-intensity laser-accelerated proton beams. Nat Phys 8, 139–142 (2012).

[b9] Daido H., Nishiuchi M. & Pirozhkov A. S. Review of laser-driven ion sources and their applications. Rep. Prog. Phys. 75, 056401 (2012). 22790586 10.1088/0034-4885/75/5/056401

[b10] Hegelich B. M. *et al.* Laser-driven ion acceleration from relativistically transparent nanotargets. New J. Phys. 15, 085015 (2013).

[b11] Macchi A., Borghesi M. & Passoni M. Ion acceleration by superintense laser-plasma interaction. Rev. Mod. Phys. 85, 751–793 (2013).

[b12] Bang W. *et al.* Temperature measurements of fusion plasmas produced by petawatt laser-irradiated D_2_-^3^He or CD_4_-^3^He clustering gases. Phys. Rev. Lett. 111, 055002 (2013). 23952411 10.1103/PhysRevLett.111.055002

[b13] Barbui M. *et al.* Measurement of the plasma astrophysical *S* factor for the ^3^He(*d*, *p*)^4^He reaction in exploding molecular clusters. Phys. Rev. Lett. 111, 082502 (2013). 24010431 10.1103/PhysRevLett.111.082502

[b14] Zylstra A. B. *et al.* Measurement of charged-particle stopping in warm dense plasma. Phys. Rev. Lett. 114, 215002 (2015). 26066441 10.1103/PhysRevLett.114.215002

[b15] Yang J. M. *et al.* Neutron production by fast protons from ultraintense laser-plasma interactions. J. Appl. Phys. 96, 6912–6918 (2004).

[b16] Roth M. *et al.* Fast ignition by intense laser-accelerated proton beams. Phys. Rev. Lett. 86, 436–439 (2001). 11177849 10.1103/PhysRevLett.86.436

[b17] Fernández J. C. *et al.* Fast ignition with laser-driven proton and ion beams. Nucl. Fusion 54, 054006 (2014).

[b18] Patel P. *et al.* Isochoric heating of solid-density matter with an ultrafast proton beam. Phys. Rev. Lett. 91, 125004 (2003). 14525369 10.1103/PhysRevLett.91.125004

[b19] Snavely R. A. *et al.* Laser generated proton beam focusing and high temperature isochoric heating of solid matter. Phys. Plasmas 14, 092703 (2007).

[b20] Brambrink E. *et al.* Direct evidence of strongly inhomogeneous energy deposition in target heating with laser-produced ion beams. Phys. Rev. E 75, 065401 (2007). 10.1103/PhysRevE.75.065401 17677318

[b21] Pelka A. *et al.* Ultrafast melting of carbon induced by intense proton beams. Phys. Rev. Lett. 105, 265701 (2010). 21231678 10.1103/PhysRevLett.105.265701

[b22] Hoarty D. J. *et al.* Equation of state studies of warm dense matter samples heated by laser produced proton beams. High Energy Density Physics 8, 50–54 (2012).

[b23] Mancic A. *et al.* Isochoric heating of solids by laser-accelerated protons: Experimental characterization and self-consistent hydrodynamic modeling. High Energy Density Physics 6, 21–28 (2010).

[b24] Mančić A. *et al.* Picosecond short-range disordering in isochorically heated aluminum at solid density. Phys. Rev. Lett. 104, 035002 (2010). 20366651 10.1103/PhysRevLett.104.035002

[b25] Dyer G. *et al.* Equation-of-state measurement of dense plasmas heated with fast protons. Phys. Rev. Lett. 101, 015002 (2008). 18764119 10.1103/PhysRevLett.101.015002

[b26] Koenig M. *et al.* Progress in the study of warm dense matter. Plasma Phys. Controlled Fusion 47, B441 (2005).

[b27] Schwoerer H. *et al.* Laser-plasma acceleration of quasi-monoenergetic protons from microstructured targets. Nature 439, 445–448 (2006). 16437110 10.1038/nature04492

[b28] Palmer C. A. J. *et al.* Monoenergetic proton beams accelerated by a radiation pressure driven shock. Phys. Rev. Lett. 106, 014801 (2011). 21231748 10.1103/PhysRevLett.106.014801

[b29] Kar S. *et al.* Ion acceleration in multispecies targets driven by intense laser radiation pressure. Phys. Rev. Lett. 109, 185006 (2012). 23215290 10.1103/PhysRevLett.109.185006

[b30] Haberberger D. *et al.* Collisionless shocks in laser-produced plasma generate monoenergetic high-energy proton beams. Nat Phys 8, 95–99 (2012).

[b31] Palaniyappan S. *et al.* Efficient quasi-monoenergetic ion beams up to 18 Mev/nucleon via self-generated plasma fields in relativistic laser plasmas. *arXiv:1506.07548* (2015). 10.1038/ncomms10170 PMC4682178 26657147

[b32] Mora P. Plasma expansion into a vacuum. Phys. Rev. Lett. 90, 185002 (2003). 12786012 10.1103/PhysRevLett.90.185002

[b33] Gittings M. *et al.* The rage radiation-hydrodynamic code. Comput. Sci. Disc. 1, 015005 (2008).

[b34] Lyon S. P. & Johnson J. D. Sesame: The los alamos national laboratory equation of state database. Los Alamos National Laboratory Report No. LA-UR-92-3407 (1992).

[b35] Palaniyappan S. *et al.* Dynamics of relativistic transparency and optical shuttering in expanding overdense plasmas. Nat Phys 8, 763–769 (2012).

[b36] Jung D. *et al.* A novel high resolution ion wide angle spectrometer. Rev. Sci. Instrum. 82, 043301 (2011). 21528999 10.1063/1.3575581

[b37] Ziegler J. F., Ziegler M. D. & Biersack J. P. Srim—the stopping and range of ions in matter (2010). Nucl. Instrum. Methods Phys. Res. B 268, 1818–1823 (2010).

[b38] Paul H. & Sánchez-Parcerisa D. A critical overview of recent stopping power programs for positive ions in solid elements. Nucl. Instrum. Methods Phys. Res. B 312, 110–117 (2013).

[b39] Boettger J., Honnell K. G., Peterson J. H., Greeff C. & Crockett S. Tabular equation of state for gold. AIP Conf. Proc. 1426, 812 (2012).

[b40] Falk K. *et al.* Equation of state measurements of warm dense carbon using laser-driven shock and release technique. Phys. Rev. Lett. 112, 155003 (2014). 24785044 10.1103/PhysRevLett.112.155003

[b41] Vinko S. M. *et al.* Creation and diagnosis of a solid-density plasma with an x-ray free-electron laser. Nature 482, 59–62 (2012). 22278059 10.1038/nature10746

[b42] Smith R. F. *et al.* Ramp compression of diamond to five terapascals. Nature 511, 330–333 (2014). 25030170 10.1038/nature13526

