# Supplementary material for: Visualization of expanding warm dense gold and diamond heated rapidly by laser-generated ion beams
Source: Sci Rep. 2015 Sep 22;5:14318. doi: 10.1038/srep14318 (PMC4585717; doi:10.1038/srep14318)
Supplement: Supplementary Information [file srep14318-s1.pdf]

Supplementary Information:
Visualization of expanding warm dense gold and diamond heated rapidly by laser-generated ion beams

W. Bang,[1] B. J. Albright,[1] P. A. Bradley,[1] D. C. Gautier,[1] S. Palaniyappan,[1] E. L. Vold,[1] M. A. Santiago Cordoba,[1] C. E. Hamilton,[1] and J. C. Fernández[1]
[1]*Los Alamos National Laboratory, Los Alamos, NM, 87544, USA*

Supplementary Fig. 1a shows the front view of the actual target on a silicon substrate. A 10 μm thick gold foil is on the right hand side of the 750 μm hole, while a 15 μm thick diamond foil is placed on the left side, separated by 156 μm. As shown in Supplementary Fig. 1b, the Al^{11+} ions are incident on the target at 45° so that a 660 nm laser beam could be sent from behind the target to probe the locations of the critical surfaces of gold and diamond. The light not blocked by gold or diamond plasma goes into the streak camera (Hamamatsu C4187), showing the edge location of gold and diamond as a function of time. The aluminum ions that penetrate the target are recorded on the magnetic ion spectrometer, which monitors shot-to-shot fluctuations in the incident ion energy spectra and fluence.

a

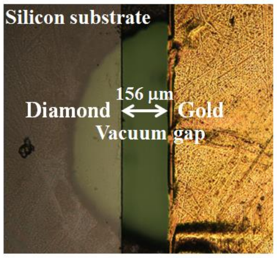

b

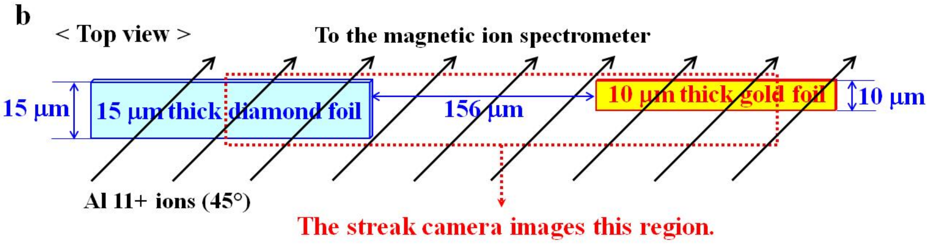

Supplementary Figure 1. Front and top view of the target. a, Front view of the target showing a diamond foil and a gold foil separated by 156 μm on the same silicon substrate with a 750 μm hole. **b,** Top view of the 15 μm thick diamond and 10 μm thick gold foils separated by 156 μm. The Al^{11+} ions (black arrows) are incident on the target at 45°, and heat the foils isochorically. These ions are recorded on the magnetic ion spectrometer after passing through the target. A probe beam backlights the target, and the streak camera images the foils expanding into the gap detecting the transmitted light.

We calculate the average absorbed energy per target atom, or heating per atom, using the following relation:

$$\text{Heating per atom} = \frac{N_{ion} < E_{deposit} >}{N_{target}}, \quad (1)$$

where N_{ion} is the total number of incident Al^{11+} ions on the target, $<E_{deposit}>$ is the average energy deposited by one Al^{11+} ion, and N_{target} is the total number of target atoms irradiated by the ion beam. Because the resulting temperatures of the plasmas were of order several eV, radiation losses are insignificant and are neglected in equation (1).

Supplementary Fig. 2 shows the calculated heating per atom from the quasi-monoenergetic Al^{11+} ion beam for gold and diamond as a function of the distance from the ion source. The heating per atom follows an inverse square law because N_{ion} is roughly proportional to 1/(source-to-target distance)^2 for target dimensions on the order of 100 μm. At the source-to-target distance of 2.37 mm, used in this experiment, each gold atom absorbs 38 (±11) eV on average from the ion beam, while a carbon atom absorbs 6.5 (±1.9) eV, corresponding to plasma temperatures of 5.1 (±1.0) eV for gold (SESAME table #2705) and 1.9 (±0.5) eV for diamond (SESAME table #2834) at solid densities according to SESAME EOS tables. Up to ±30% fluctuation in the ion fluence was assumed for the error calculations.

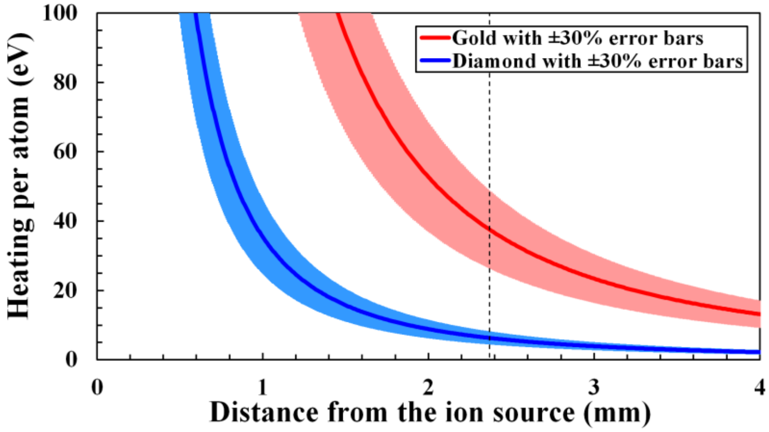

Supplementary Figure 2. Heating per atom from the aluminum ion beam for gold and diamond versus the source-to-target distance. A vertical dashed line indicates the distance (2.37 mm) used in this experiment.

Supplementary Fig. 3 shows a streaked image where diamond turns opaque immediately after heating. Prior to the isochoric heating, a diamond foil transmits 69% of the 660 nm optical probe light. As the foil is heated by the aluminum ions (~20 ps rise time), the electron density quickly becomes about 6×10^{22} cm^{-3} ($\langle Z\rangle\sim0.3$) far exceeding the critical density of 2.56×10^{21} cm^{-3} for the 660 nm light, and the diamond plasma blocks the probe light. Even after expanding into a vacuum for 5 ns, the diamond plasma still remains opaque because the electron density exceeds the critical density. A crude estimate of the average electron density based on the measured expansion speed in Fig. 4 shows that it drops down to 1×10^{22} cm^{-3} after 5 ns.

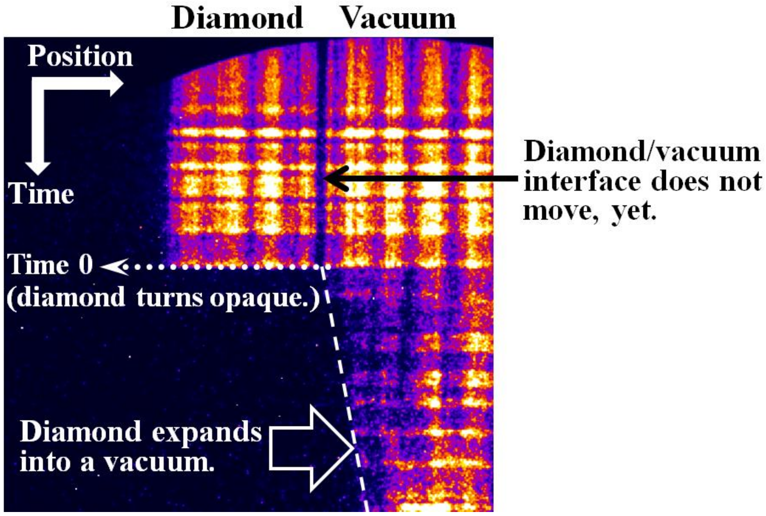

Supplementary Figure 3. Another streak camera image of an expanding diamond into a vacuum. The diamond foil is on the left side of the hole on this shot. At time 0, the quasi-monoenergetic aluminum ions heat diamond isochorically, triggering its expansion into a vacuum. After heating, the electron density of diamond increases very quickly beyond the critical density, and the 660 nm optical probe light cannot penetrate through diamond.
